# Evaluation of a cyberbullying prevention program in elementary schools: The role of self-esteem enhancement

**DOI:** 10.3389/fpsyg.2022.980091

**Published:** 2022-09-23

**Authors:** Thanos Touloupis, Christina Athanasiades

**Affiliations:** Department of Psychology, Aristotle University of Thessaloniki, Thessaloniki, Greece

**Keywords:** cyberbullying, prevention, elementary school students, special educational needs, self-esteem

## Abstract

Although elementary schools are considered a fertile ground for promoting positive behaviors among students (such as safe online practices), to date, almost no study has examined the effectiveness of a cyberbullying prevention program among elementary school students of typical and non-typical development. The present study evaluated the effectiveness of such a school-based European funded preventive program (TABBY, Threat Assessment of Bullying Behavior in Youth) among sixth graders with and without special educational needs (SEN). The study also examined the predictive role of self-esteem in students’ cyberbullying involvement. Overall, 240 students from randomly selected Greek schools completed a self-report questionnaire, which included a scale on cyberbullying and self-esteem. Following an experimental longitudinal research design, the intervention was applied to the experimental (*N* = 120) but not to the control group of students (*N* = 120). Each group consisted of both students with (*N* = 60) and without SEN (*N* = 60). The evaluation was based on the completion of the self-report questionnaire before (1^st^ phase), immediately after (2^nd^ phase), and 6 months after the intervention was completed (3^rd^ phase) by trained general and special education teachers. According to the findings, students’ cyberbullying engagement (as bullies/victims) decreased significantly in the second and third phase, and especially for those with SEN. Additionally, self-esteem negatively predicted students’ involvement in cyberbullying (as bullies/victims) in all three phases. The findings partially support the appropriateness of interventions within the elementary school context in order to enhance self-esteem and promote a safe online culture among students of typical as well as atypical development.

## Introduction

Today the easy and instant access of youths to new technologies has highlighted the phenomenon of cyberbullying as a common online risk behavior among them who intentionally use electronic devices to harm others (Smith et al., 2008), with a negative impact at both the socio-emotional and the educational level (Rudnicki et al., 2022).

International findings show that cyberbullying concerns not only adolescents (Ng et al., 2022) but also elementary school students (DePaolis and Williford, 2015; Aizenkot and Kashy-Rosenbaum, 2018). This has been more obvious during the last 2 years of the COVID-19 pandemic since social isolation has led to the dominance of the internet in youths’ daily lives (Chen et al., 2022). More specifically, according to relevant data, elementary school students are involved in cyberbullying either as bullies or as victims in percentages up to 8 and 12%, respectively (DePaolis and Williford, 2015; Zhang et al., 2021).

Furthermore, students with special educational needs (SEN) are also involved in cyberbullying, sometimes even more than students of typical development. Specifically, students with learning disabilities (LD), autistic spectrum disorder (ASD), and attention deficit hyperactivity disorder (ADHD), due to their learning and behavioral difficulties, usually experience labelling issues and stigmatization at school. Therefore, they are considered a vulnerable group for engaging in bullying incidents, not only in the physical context but also in cyberspace, reaching percentages of 13.5% for bullies and 23.5% for victims (Aslan, 2016; Jenaro et al., 2018; Wright and Wachs, 2020; Touloupis and Athanasiades, 2022).

Apart from SEN, individual emotional characteristics have also been highlighted as significant predictive factors for cyberbullying involvement (either as victims or as bullies). Self-esteem, namely someone’s evaluative self-perspective (Leontari, 1996), has consistently proved to be one of these factors. Related findings show that both students with and without SEN when experiencing low self-esteem (e.g., due to their learning/behavioral difficulties or other reasons) are more likely to seek peer support and acceptance, even in cyberspace (Kokkinos and Panayiotou, 2004; Lei et al., 2020).

The above data confirm the necessity to implement cyberbullying prevention programs at schools (particularly during the elementary school years) that enhance students’ emotional skills. The implementation of these programs is based on Bronfenbrenner’s (1989) ecological systems theory, which proposes that at micro-environmental levels, such as the school environment, effective practices can be applied to influence students’ behaviors and attitudes. Consequently, following a holistic context-based approach, prevention programs encompass the involvement of all stakeholders in the school community (e.g., students, teachers; Jabulani and Edward, 2021).

In general, teachers’ willingness to get involved and apply an intervention depends on the organizational characteristics and working circumstances of the school context, which are usually different in secondary and elementary school contexts (at least in Greece). For example, in elementary schools participatory decision-making processes, peer mentorship, and collaborative practices are widely used at both the school and classroom level, contributing to a more positive and creative school climate as well as to closer interpersonal relationships within the school community (Wong et al., 2008; Ζapata-Caceres et al., 2021). In this context, teachers feel more comfortable in having students express their thoughts, perceptions, and enthusiasm about technological devices, enhancing in this way teachers’ vigilance towards students’ unsafe patterns of online behavior (Wong et al., 2008; Touloupis and Athanasiades, 2020a; Ζapata-Caceres et al., 2021). Additionally, the emphasis placed on students’ technological literacy from the beginning of elementary education (Vélez and Zuazua, 2017; Touloupis and Athanasiades, 2018, 2020b) offers more opportunities for teachers to raise issues of ethical and safe online behavior. Finally, elementary school teachers, due to their familiarity with critical issues related to educational/school psychology during their undergraduate studies (Katman and Tutkun, 2015), are more likely to motivate students to engage in positive behaviors, even in cyberspace, and to act as role models for children.

Therefore, it is deduced that teachers, especially in the elementary school context, can play a vital role in the implementation of cyberbullying prevention programs. The international literature highlights a limited number of studies evaluating such programs (e.g., “I-SAFE Program,” “Missing Program,” “HAHASO study,” “Let us Fight It Together”), which are aimed almost exclusively at the school community of secondary education (Mishna et al., 2009; Gaffney et al., 2019). The results of these studies seem to be contradictory since most of the programs, although promoting adolescents’ knowledge of safe internet use, do not reduce adolescents’ involvement in cyberbullying, implying a limited effectiveness of the programs (Mishna et al., 2009; Thompson et al., 2013). On the other hand, other programs, such as the “ConRed Cyberbullying Prevention Program” and the “Media Heroes,” have proved effective in increasing adolescents’ safe online behavior and reducing their involvement in cyberbullying (Ortega Ruiz et al., 2012; Schultze-Krumbholz et al., 2018). Furthermore, it worth noting that in some of the above programs, the implementation was carried out by psychologists or other mental health specialists (Mishna et al., 2009), even though teachers have been proposed as key figures in schools for the effective implementation of prevention programs (Yoon and Bauman, 2014). This could explain, to some extent, the contradictory findings regarding the effectiveness of cyberbullying prevention programs.

In Greece, the TABBY program (threat assessment of bullying behavior in youth) for the prevention of cyberbullying among adolescents reflects a scientifically integrated effort to develop and implement such an action of European standards. This program has been successfully implemented in Greek high schools, thereby reducing the percentages of adolescents who have been victims of cyberbullying (Athanasiades et al., 2015). However, considering that the program, compared to cyber-victims, did not reduce the rates of Greek adolescents who acted as cyberbullies, it is implied that there may be a need to enrich the content of the TABBY program. For example, based on the reported negative association between low self-esteem and cyberbullying behavior among students with and without SEN (Kokkinos and Panayiotou, 2004; Touloupis and Athanasiades, 2022), it is likely that within the program emphasis should be given not only to students’ cyberbullying awareness and sensitization but also to the enhancement of their emotional skills, such as self-esteem, as a protective filter against the phenomenon. Additionally, although elementary school students, and mainly those with SEN, are considered equally vulnerable to involvement (either as victims or as bullies) in cyberbullying as adolescents (Wright and Wachs, 2020; Zhang et al., 2021), there is no scientific documentation for the appropriateness of the TABBY program in this student population.

The above literature highlights the necessity to implement and evaluate the effectiveness of a cyberbullying prevention program, such as TABBY, in the elementary school context, which can be beneficial for both students with and without SEN. Also, the study intends to investigate the role of students’ self-esteem in their cyberbullying involvement. Specifically, the research goals were to investigate (a) the effect of the intervention on students with and without SEN in relation to their cyberbullying involvement before, immediately after the intervention, and 6 months later, and (b) the predictive role of self-esteem in cyberbullying involvement in the three phases of the study (before, immediately after, and 6 months after the intervention) for students with and without SEN.

The corresponding research hypotheses were the following:

Hypothesis 1 (H1)

It was speculated that the intervention will be effective immediately after and 6 months after the intervention was completed for both students without (H1a) and with SEN (H1b).

Hypothesis 2 (H2)

It was speculated that self-esteem of both students with and without SEN will negatively predict their cyberbullying involvement in all three phases of the study (Kokkinos and Panayiotou, 2004; Lei et al., 2020; Hypothesis 2).

## Materials and methods

### Sample

The participants were 240 sixth grade^1^ students (*N* = 120 with SEN and *N* = 120 without SEN), who had internet access and made use of social media (e.g., Facebook, Instagram). Almost half of the students with SEN (*N* = 59 [49.2%]) and without SEN (*N* = 65 [54.2%]) were boys, while their age ranged between 11 and 12 years (*Mean* = 11.8, *SD* = 0.45). The students came from 29 randomly selected general education elementary schools from the city area of Thessaloniki (the second largest Greek city after Athens). All schools had integration classrooms in which students with SEN were taught by special education teachers daily. The students with SEN had been formally diagnosed in the past with LD (e.g., dyslexia; *N* = 45 [37.5%]), ASD of high-functioning (previously known as Asperger syndrome according to DSM-IV; *N* = 39 [32.5%]) and ADHD (*N* = 36 [30%]), and attended for a few hours every day the general education classrooms, as they had (at least) a normal Intelligent Quotient (IQ) and could meet their classroom curriculum (MINEDU, 2018). In the pilot study participated 54 sixth grade students (*N* = 27 with SEN and *N* = 27 without SEN). However, the pilot administration of the questionnaires did not indicate the need to be modified. Consequently, the pilot sample was consolidated with the main sample (*N* = 186), resulting in the total sample of the study (*N* = 240).

### Questionnaires

Apart from answering to demographic questions (e.g., gender, age), participants completed the following two self-reported questionnaires:

*Cyberbullying questionnaire*: Cyberbullying experiences were investigated through a short version of the “Cyberbullying Questionnaire” (Smith et al., 2006), which examines the four frequently reported cyberbullying behaviors among elementary school students (sending text messages, spreading rumors, circulating audiovisual material, and making online calls; Touloupis and Athanasiades, 2014) with questions such as the following: “Have you spread, in the last year, negative rumors or comments about someone on social media (e.g., Facebook, Instagram, Twitter) to make him/her feel bad/sad/upset?” Questions were answered on a five-point scale (from 1 = *I have not done anything similar/Nothing similar has ever happened to me* to 5 = *I do it/It happens to me several times a week*).

According to previous studies (Touloupis and Athanasiades, 2014) the questionnaire reflects two factors, online victimization and online bullying. A confirmatory factor analysis, using the Maximum Likelihood method, was applied and confirmed the above two-dimensional model, which had a very good fit, *χ*^2^(93, *N* = 240) = 139.098, *p* < 0.05, CFI = 0.942, TLI = 0.951, RMSEA = 0.039, SRMS = 0.034. Two factors emerged with eigenvalue >1.0 and significant interpretive values: Factor 1 = Online victimization, explaining 39.11% of the total variance, and Factor 2 = Online bullying, explaining 28.03% of the total variance. The internal consistency indexes were satisfactory: Factor 1 (*α* = 0.811) and Factor 2 (*α* = 0.799).

*Self-esteem scale*: Students’ self-esteem was investigated with the Greek version (Kokkiades and Kourkoutas, 2016) of Rosenberg’s “Self-esteem Scale” (Rosenberg, 1989), which includes 10 proposals (e.g., “I take a positive attitude toward myself”) examining the way people feel about themselves and forming a single factor (“Self-esteem”). Proposals were answered on a five-point Likert scale (from 1 = *Strongly disagree* to 5 = *Strongly agree*).

A confirmatory factor analysis, using the Maximum Likelihood method, was applied and confirmed the unidimensional model, which had a very good fit, *χ*^2^(88, *N* = 240) = 231.128, *p* < 0.05, CFI = 0.949, TLI = 0.941, RMSEA = 0.037, SRMS = 0.038. The single-factor model had eigenvalue >1.0 and significant interpretive value: Factor 1 = Self-esteem, explaining 52.11%. The internal consistency index was satisfactory (*α* = 0.889).

### Procedure

Once the Greek Ministry of Education approved the study, the researchers informed the selected schools and the students’ parents/legal guardians regarding the purpose and the procedure of the study. Following an experimental longitudinal research design, the students from all schools were divided into an experimental and a control group. Each group included students with SEN (*N* = 60) and without SEN (*N* = 60). In the pretest phase of the study both groups completed the questionnaires in the classrooms. Subsequently, an intervention based on the TABBY program (Athanasiades et al., 2015), which was enriched with experiential activities and material for the enhancement of students’ self-esteem, was applied only to the experimental group by the specially trained general and special education teachers from the selected schools. Their nine-hour seminar training addressed the importance of holistic/systemic school interventions that focus on critical contextual factors (e.g., classroom climate, interpersonal relationships, collaborative practices) to bring about positive changes in students’ behavior and emotions. The intervention in the classrooms lasted 4 hours and included audiovisual material regarding different forms of cyberbullying, a discussion on related legal issues and the role of schools in countering cyberbullying, as well as experiential activities to enhance self-esteem. For the comparative evaluation of the short- and long-term effectiveness of the intervention, students completed again the questionnaires 2 weeks (1^st^ post-test) and 6 months after the intervention was completed (2^nd^ post-test). Students and teachers participated in the study voluntarily, and the anonymity of the data was preserved.

## Results

### Evaluation of the effectiveness of the intervention on experimental and control group

To examine the effectiveness of the intervention immediately after (2^nd^ phase) and 6 months after its completion (3^rd^ phase) repeated measures ANOVA was used. The intervention seemed to affect statistically significantly students’ online victimization, Pillai’s Trace = 0.129, *F*(3, 237) = 5.887, *p* < 0.001, partial *η*^2^ = 0.431, and online bullying, Pillai’s Trace = 0.208, *F*(3, 237) = 11.231, *p* < 0.001, partial *η*^2^ = 0.401. Violation of the Sphericity assumption of Mauchly’s *W* (*p* < 0.05) led to Huynh-Feldt’s correction of degrees of freedom in cases of online victimization, *F*(2.8, 301.44) = 8.423, *p* < 0.001*,* partial *η*^2^ = 0.401, and online bullying, *F*(2.9, 411.91) = 10.989, *p* < 0.001, partial *η*^2^ = 0.398.

Pairwise comparisons among the phases of the study, applying the Bonferroni criterion (*p* < 0.017), showed statistically significant differences concerning students’ online victimization and online bullying before, immediately after and 6 months after the intervention. Based on Table 1, compared to control group, experimental groups’ involvement in cyberbullying either as victims (online victimization) or as bullies (online bullying) significantly decreased immediately after and 6 months after the intervention.

**Table 1:** Evaluation of the effectiveness of the intervention regarding online victimization/bullying.

	Experimental group (*N* = 120)						Control group (*N* = 120)					
	Before the intervention		Immediately after the intervention		Six months after the intervention		Before the intervention		Immediately after the intervention		Six months after the intervention	
	Mean	S.D.	Mean	S.D.	Mean	S.D.	Mean	S.D.	Mean	S.D.	Mean	S.D.
Online victimization	3.09	1.23	2.43	1.12	2.45	1.97	3.11	1.82	3.02	0.93	3.08	1.03
Online bullying	3.06	1.09	2.49	1.08	2.39	1.09	3.01	1.22	2.98	1.04	3.02	1.29

S.D.: standard deviation.

### Differences between students with SEN and without SEN of the experimental group regarding cyberbullying involvement

Focusing on the experimental group, *T*-test for independent groups was applied to examine differences in the effectiveness of the intervention between students with (*N* = 60) and without SEN (*N* = 60). There were statistically significant differences between the above two subgroups regarding online victimization and online bullying immediately after/2^nd^ phase (online victimization: *t*(117) = 2.546, *p* = 0.008, online bullying: *t*(137) = 3.119, *p* = 0.010) and 6 months after the intervention/3^rd^ phase (online victimization: *t*(117) = 8.304, *p* = 0.011, online bullying: *t*(137) = 11.201, *p* = 0.031). Specifically, students with SEN were statistically less involved in cyberbullying either as victims or as bullies, compared to students without SEN in the 2^nd^ (victims: students with SEN [*Mean* = 2.49, *SD* = 2.01] vs. students without SEN [*Mean* = 2.89, *SD* = 2.12], bullies: students with SEN [*Mean* = 2.23, *SD* = 1.93] vs. students without SEN [*Mean* = 2.62, *SD* = 2.09]) and 3^rd^ phase of the study (victims: students with SEN [*Mean* = 2.44, *SD* = 2.05] vs. students without SEN [*Mean* = 2.91, *SD* = 1.89], bullies: students with SEN [*Mean* = 2.21, *SD* = 2.10] vs. students without SEN [*Mean* = 2.65, *SD* = 2.11]).

### Effect of the type of SEN on online victimization/bullying for students of the experimental group

Furthermore, to investigate differences on students’ online victimization/bullying based on their type of SEN (learning disabilities, ASD, ADHD), in the three phases of the study, MANOVAs analyses were applied. In all phases the required assumptions were met: 1^st^ phase [Box’s Test of Equality of Covariance Matrices: Box’s *M* = 192.11, *F* = 3.31, *p* = 0.08, and Levene’s Test of Equality of Error Variances for online bullying (*F* = 2.48, *df*1 = 3, *df*2 = 236, *p* = 0.31) and online victimization (*F* = 2.83, *df*1 = 3, *df*2 = 236, *p* = 0.41)], 2^nd^ phase [Box’s Test of Equality of Covariance Matrices: Box’s *M* = 181.21, *F* = 4.05, *p* = 0.10, and Levene’s Test of Equality of Error Variances for online bullying (*F* = 3.89, *df*1 = 3, *df*2 = 236, *p* = 0.23) and online victimization (*F* = 2.44, *df*1 = 3, *df*2 = 236, *p* = 0.11)], and 3^rd^ phase [Box’s Test of Equality of Covariance Matrices: Box’s *M* = 201.44, *F* = 3.15, *p* = 0.29, and Levene’s Test of Equality of Error Variances for online bullying (*F* = 2.09, *df*1 = 3, *df*2 = 236, *p* = 0.39) and online victimization (*F* = 2.32, *df*1 = 3, *df*2 = 236, *p* = 0.14)].

The MANOVAs results showed that immediately after (2^nd^ phase) and 6 months after the intervention (3^rd^ phase), there was a significant interaction effect of the type of SEN on students’ involvement in cyberbullying: 2^nd^ phase (Pillai’s Trace = 0.052, *F*(3, 236) = 3.209, *p* < 0.001, partial *η*^2^ = 0.39), and 3^rd^ phase (Pillai’s Trace = 0.083, *F*(3, 236) = 9.943, *p* < 0.001, partial *η*^2^ = 0.41). Also, in these two phases, the above effect proved significant for both cyberbullying roles: 2^nd^ phase (online victimization, *F*(1, 238) = 9.332, *p* < 0.001, partial *η*^2^ = 0.49, online bullying, *F*(1, 238) = 8.320, *p* < 0.001, partial *η*^2^ = 0.44), and 3^rd^ phase (online victimization, *F*(1, 238) = 8.459, *p* < 0.001, partial *η*^2^ = 0.38, online bullying, *F*(1, 238) = 11.298, *p* < 0.001, partial *η*^2^ = 0.34).

The direction of this effect on the above cases is presented in Table 2, showing that in immediately after (2^nd^ phase) and 6 months after the intervention (3^rd^ phase) students with ASD and ADHD of the experimental group were involved in cyberbullying as victims and bullies to a relatively lesser extent (their lower Means are in bold), compared to students with learning disabilities of the experimental group.

**Table 2:** Effect of the type of SEN on online victimization/bullying for students of the experimental group in the three phases of the study.

Phases of the study		Type of SEN	Mean	S.D.
Before the intervention	Online victimization	Learning disabilities	3.11	0.79
		Asperger syndrome	3.07	0.52
		ADHD	3.08	0.49
	Online bullying	Learning disabilities	3.07	0.88
		Asperger syndrome	3.05	0.89
		ADHD	3.06	0.43
Immediately after the intervention	Online victimization	Learning disabilities	2.67	0.72
		Asperger syndrome	**2.30**	0.59
		ADHD	**2.31**	0.89
	Online bullying	Learning disabilities	2.74	0.87
		Asperger syndrome	**2.39**	0.94
		ADHD	**2.36**	0.82
Six months after the intervention	Online victimization	Learning disabilities	2.69	0.72
		Asperger syndrome	**2.32**	0.59
		ADHD	**2.34**	0.89
	Online bullying	Learning disabilities	2.57	0.47
		Asperger syndrome	**2.29**	0.84
		ADHD	**2.30**	0.92

S.D.: standard deviation.

### Correlations between cyberbullying and self-esteem

To examine the pattern of correlations among the variables involved for the total sample in the three phases of the study, Pearson (Pearson *r*) correlations were applied. It was found that before the intervention (1^st^ phase), self-esteem negatively predicted students’ online victimization (*r* = −0.329, *p* < 0.01) and online bullying (*r* = −0.311, *p* < 0.01). Stronger negative predictive correlations were found between self-esteem and the two roles of cyberbullying immediately after (2^nd^ phase [victims: *r* = −0.455, *p* < 0.01, bullies: *r* = −0.409, *p* < 0.01]), and 6 months after the intervention (3^rd^ phase [victims: *r* = −0.441, *p* < 0.01, bullies: *r* = −0.432, *p* < 0.01]).

### The predictive role of self-esteem in cyberbullying

The predictive relationship between students’ self-esteem and their online victimization/bullying was checked through linear regressions. Although, the regression indexes of *R*^2^ were generally low, comparing the standardized regression coefficients (Table 3) for the three phases of the study, there were found stronger negative predictive relationships between the above variables for the experimental group immediately after (2^nd^ phase; see the corresponding bold indexes in Table 3) and 6 months after the intervention (3^rd^ phase; see the corresponding bold indexes in Table 3), compared to the 1^st^ phase (before intervention).

**Table 3:** The predictive role of self-esteem in cyberbullying for experimental and control group in the three phases of the study.

Three phases of the study	Predictive factor	Cyberbullying involvement	Groups	*R* ^2^	*β*	*t*	*p*
Before the intervention	Self-esteem	Online victimization	Experimental	0.033	−0.210	−4.330	0.032
			Control	0.031	−0.221	−4.442	0.039
		Online bullying	Experimental	0.052	−0.280	−3.989	0.015
			Control	0.045	−0.229	−4.032	0.023
Immediately after the intervention	Self-esteem	Online victimization	Experimental	**0.065**	−**0.521**	**−8.732**	**0.009**
			Control	0.024	−0.242	−5.301	0.032
		Online bullying	Experimental	**0.081**	−**0.449**	**−7.887**	**0.005**
			Control	0.048	−0.201	−4.911	0.029
Six months after the intervention	Self-esteem	Online victimization	Experimental	**0.061**	−**0.489**	**−7.439**	**0.004**
			Control	0.029	−0.188	−3.773	0.022
		Online bullying	Experimental	**0.077**	−**0.501**	**−7.014**	**0.015**
			Control	0.033	−0.302	−4.209	0.042

*β*: standardized regression coefficient.

## Discussion

The study evaluated the effectiveness of a cyberbullying intervention based on the TABBY program in the elementary school environment where students with SEN and without SEN co-exist, examining at the same time the role of self-esteem in their cyberbullying involvement. According to the results, the intervention generally proved effective as the experimental group (students with and without SEN) reported lower rates of cyberbullying involvement (either as victims or as bullies) not only immediately after but also 6 months after the intervention was completed. This is in line with H1a and H1b, highlighting the short-term and the long-term effectiveness, respectively, of the intervention not only for victims, as was found before (Athanasiades et al., 2015), but also for bullies of typical development. Given the different contextual characteristics of elementary schools compared to high schools (i.e., closer interpersonal relationships, sense of belonging, less competitive school climate; Ζapata-Caceres et al., 2021), it could be inferred that a cyberbullying intervention implemented in such a context could benefit all students, even the perpetrators of such aggressive behaviors. After all, it has been reported that in this setting teachers are also more likely to become involved and committed to the effective implementation of the intervention (Wong et al., 2008).

Furthermore, when adopting an intragroup comparative perspective within the experimental group, it seemed that students with SEN benefitted more from the intervention, compared to their peers without SEN. This could be attributed to the fact that the intervention was implemented not only by general education teachers but also by special education colleagues. The latter collaborate closely with the general education teachers and spend many hours daily with students with SEN in the integration classrooms of elementary schools; they are usually well trained in the implementation of interventions for the SEN (MINEDU, 2018), and consequently, special education teachers may contribute significantly to the effective implementation of the intervention. The above parameters are likely to have made students, and especially those with SEN, report lower involvement in cyberbullying (either as victims or as bullies) immediately after and 6 months after the intervention. Also, focusing only on students with SEN, it was found that those with ASD and ADHD experienced a relatively higher benefit from the intervention compared to their peers with learning disabilities (e.g., dyslexia). Considering that learning audiovisual material (e.g., videos, pictures) has proved very helpful mainly for students with social and behavioral difficulties, such as those with ASD and ADHD (Rogers, 2013), we could justify that cyberbullying awareness through corresponding material was slightly greater for these students in the second and the third phase of the study.

The above parameters, along with the fact that in secondary education students with SEN are not supported by the same teacher (as a stable key figure) but by different specialties of teachers for fewer hours daily (MINEDU, 2018), could partially support the fact that the elementary school context may act as a fertile ground/organization for applying appropriate strategies in order for students of typical and non-typical development to benefit from preventive actions. Nevertheless, as the TABBY program has not been implemented to date in secondary school students with SEN, it would be worth conducting similar study to elicit “comparative” findings regarding the appropriateness of a secondary school environment to promote related prevention actions for this student population.

Furthermore, in line with hypothesis 2 and other related findings (Kokkinos and Panayiotou, 2004; Lei et al., 2020), the results revealed a negative predictive role of self-esteem in cyberbullying involvement (either as victims or as bullies) in all three phases of the study, although with low predictive values. Considering the experiential activities during the intervention for the enhancement of students’ self-esteem, we could explain the fact that this predictive relationship seemed to be stronger immediately after and 6 months after the intervention was completed for the experimental group. Based on the fact that young children’s socio-emotional skills are gradually shaped from the early years of their school attendance (Dowling, 2014), we could view the elementary school context as a fertile ground for strengthening longitudinally children’s self-esteem as a protective factor against cyberbullying. Finally, considering the reported correlation between low self-esteem and online perpetrators’ behavior (Patchin and Hinduja, 2010), we could argue that the intervention’s emphasis on enhancing students’ self-esteem may have contributed to the decreased rates not only of cyber-victims but of cyberbullies as well in the present study. This is a finding that was not the case when the intervention was implemented in secondary education without self-esteem activities included (Athanasiades et al., 2015). Nevertheless, future related studies based on the TABBY program should examine the extent of the predictive role of self-esteem in elementary school students’ involvement in cyberbullying.

In conclusion, it seems that a cyberbullying intervention based on the TABBY program could be effectively implemented in the elementary school context, which is identified with the sensitive years of students’ socio-emotional development as well as with specific organizational characteristics (e.g., close collaboration between teachers of general and special education). In this context, it seems that key figures, such as teachers of general and special education, may effectively enhance emotional skills and subsequently bring about positive changes in the cyber behavior of students of typical and non-typical development. Undoubtedly, future related studies in elementary schools could confirm and extend the above findings.

## Data availability statement

The datasets presented in this article are not readily available because due to the specificity of the sample and the sensitive nature of the research topic, the participating students and parents/guardians were assured raw data and material would remain confidential and would not be shared. Requests to access the datasets should be directed to touloupis@psy.auth.gr.

## Ethics statement

The studies involving human participants were reviewed and approved by Greek Ministry of Education, Institute of Educational Policy. Written informed consent to participate in this study was provided by the participants’ legal guardian/next of kin.

## Author contributions

TT conducted the study, performed the statistical analyses, and wrote the research article. CA supervised the whole procedure. All authors contributed to the article and approved the submitted version.

## Conflict of interest

The authors declare that the research was conducted in the absence of any commercial or financial relationships that could be construed as a potential conflict of interest.

## Publisher’s note

All claims expressed in this article are solely those of the authors and do not necessarily represent those of their affiliated organizations, or those of the publisher, the editors and the reviewers. Any product that may be evaluated in this article, or claim that may be made by its manufacturer, is not guaranteed or endorsed by the publisher.

## Footnotes

^1^Greek students’ attendance in elementary school lasts 6 years.
